# Modulation of Hippocampal Neural Plasticity by Glucose-Related Signaling

**DOI:** 10.1155/2015/657928

**Published:** 2015-04-21

**Authors:** Marco Mainardi, Salvatore Fusco, Claudio Grassi

**Affiliations:** Institute of Human Physiology, Medical School, Università Cattolica, Largo Francesco Vito 1, 00168 Rome, Italy

## Abstract

Hormones and peptides involved in glucose homeostasis are emerging as important modulators of neural plasticity. In this regard, increasing evidence shows that molecules such as insulin, insulin-like growth factor-I, glucagon-like peptide-1, and ghrelin impact on the function of the hippocampus, which is a key area for learning and memory. Indeed, all these factors affect fundamental hippocampal properties including synaptic plasticity (i.e., synapse potentiation and depression), structural plasticity (i.e., dynamics of dendritic spines), and adult neurogenesis, thus leading to modifications in cognitive performance. Here, we review the main mechanisms underlying the effects of glucose metabolism on hippocampal physiology. In particular, we discuss the role of these signals in the modulation of cognitive functions and their potential implications in dysmetabolism-related cognitive decline.

## 1. Introduction

Glucose homeostasis is controlled by an intricate network composed of organs, glands, and molecular messengers, whose primary aim is to maintain an optimal balance between energy stores and immediately available fuel for cellular processes. Hence, it is not surprising that the brain is endowed with mechanisms for sensing glucose levels [1]. In addition, cerebral areas such as the basal hypothalamus and the brainstem contain neuronal populations which act as controllers of physiological and behavioral reactions (i.e., regulation of feeding behavior) in response to oscillations in glucose levels and bodily energy demands [2].

**Figure 1:**
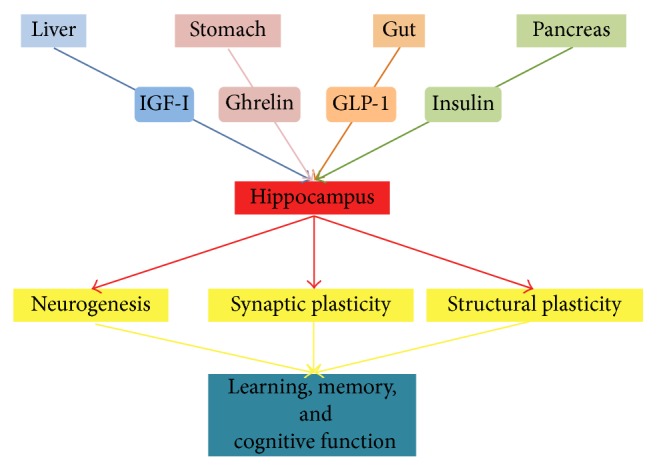
Schematic showing the convergence of the action of key molecules in metabolic signaling on different aspects of hippocampal physiology.

Strikingly, glucose sensing in the brain appears to be also involved in the modulation of brain cell functions having no direct relationships with metabolism. Indeed, glucose-related signaling has a strong impact on neuronal activity. In particular, we will here attempt to review the increasing body of evidence indicating that messengers essential to glucose homeostasis also affect at multiple levels the activity of the hippocampus, which is a brain area critically involved in cognitive functions. We will focus on a few key molecules: insulin and insulin-response substrates (IRSs), insulin-like growth factor-I (IGF-I), glucagon-like peptide-1 (GLP-1), and ghrelin. After briefly summarizing nonmetabolic glucose-related signaling in the brain, we will try to convey the message that these molecules exert multiple actions on hippocampal physiology by affecting structural and functional neuroplasticity. This is, in turn, correlated to modifications in hippocampal-dependent learning and memory processes (Figure 1).

Finally, we will give an overview of the relevance of these phenomena for pathology, since the involvement of metabolic dysregulation in neuronal function impairment is an emerging topic with promising translational implications.

## 2. Outlines of Glucose Homeostasis-Related Signals Acting on the Hippocampus

In response to physiological stimuli and environmental conditions, the central nervous system undergoes structural and functional changes, both during development and throughout adulthood. This process of “plasticity” involves neurogenesis, that is, proliferation and differentiation of neural stem cells (NSCs), as well as changes in the morphology and activity of differentiated neurons. These adjustments are instrumental to the brain orchestration of various peripheral organs functions, in order to adapt energy expenditure to nutrient availability. In this regard, the hypothalamus-pituitary axis integrates humoral signals and coordinates behavioral and metabolic responses of the whole body [3]. However, the hypothalamus is only one of the brain areas sensitive to hormones and metabolic signals. Indeed, food seeking during fasting is a complex activity which involves information processing to identify or remember the location of resources necessary for survival.

In keeping with this, brain areas not involved in feeding control synthesize receptors for insulin, insulin-like peptides such as insulin-like growth factor-I (IGF-I), glucagon-like peptide-1 (GLP-1), and ghrelin [4, 5]. Accordingly, the activity of neural circuits in the hippocampus is influenced by metabolic stimuli and energy supply [6].

Moreover, neurons are high energy-consuming cells and their function is markedly affected by the energy status. In the brain, most energy is consumed to generate action potentials and postsynaptic potentials [7, 8]. Additionally, glucose metabolism provides energy for the biosynthesis of neurotransmitters in differentiated neurons [9] and for NSC fate determination [10]. Importantly, the astrocytic energy sources glycogen [11] and lactate [12] seem to be directly relevant for learning and memory, although the underlying mechanisms have not yet been elucidated.

Finally, the transporters GLUT1 and GLUT3 mediate glucose uptake from extracellular fluid into glial and neuronal cells, respectively [13]. GLUT1 and GLUT3 are insulin-independent transporters, suggesting that the impact of insulin and related signals on brain plasticity should be independent of glucose uptake.

In addition to a direct effect of glucose levels on neuronal metabolism, hormones involved in glucose homeostasis activate different signal transduction cascades in the brain. This process results in effects which go well beyond the regulation of neuronal energy demand and metabolism (see below). Indeed, insulin and IGF-I activate the phosphatidylinositol trisphosphate kinase (PI3K)/Akt and Ras/MAPK-ERK pathways, thus affecting gene expression, with huge consequences for NSC proliferation and neuronal activity [14, 15].

GLP-1 is secreted by the gut in response to satiation and participates in glucose homeostasis. Indeed, it was first characterized for its ability to enhance insulin release from pancreatic *β*-cells, thus leading to increased glucose sensitivity [16]. Subsequent research in rodents has shown that GLP-1 receptors are also present on neurons, with intense expression in the CA hippocampal region [17, 18], and that their activation stimulates the activity of the MAP kinase pathway [19].

On the other hand, the stomach secretes ghrelin, which stimulates feeding behavior [20], and exerts a global counterregulatory action in comparison to insulin [21]. In the brain, ghrelin binds the growth hormone secretagogue receptor 1a (GHS-R1a) and controls the G protein-mediated activation of the PI3K/Akt, Ras/MAPK-ERK, and PKA/CREB pathways [22–24].

In addition, it is interesting to notice the close similarity between the intracellular signaling pathways activated by glucose metabolism regulators and those controlled by neurotrophins [25]. This convergence emphasizes the importance of metabolic mediators for proper functioning of neural circuits.

In particular, the cAMP-responsive element binding (CREB) transcription factor has been largely studied as mediator of neurotrophin-triggered neuronal differentiation, survival, and plasticity in the brain, and it has been characterized as metabolic sensor modulated by nutrient depletion and fasting hormones [26, 27]. Calorie restriction also induces the expression of the NAD^+^-dependent histone deacetylase Sirtuin 1 (SIRT1), which has been recently identified as a partner in CREB-dependent gene expression, thus highlighting a novel mechanism linking metabolic homeostasis and brain health [28]. In a mouse model of brain insulin resistance, intracerebral injection of streptozotocin reduces the activity of SIRT1 and causes cognitive impairment, an alteration prevented by administration of the SIRT1 activator resveratrol [29]. Indeed, SIRT1 promotes the CREB-dependent expression of* Brain-Derived Neurotrophic Factor* (*BDNF*) and other neuroprotective genes [30]. Moreover, CREB-dependent transactivation of genes regulating neuronal survival, metabolism, and plasticity (like PGC1*α* and nNOS) in calorie-restricted mice requires SIRT1 [31]. In keeping with these data, electrophysiological and cognitive brain responses to calorie restriction are similarly impaired in mice harboring brain-specific inactivation of SIRT1 or CREB [32]. Finally, in the mouse hippocampus, SIRT1 transcription is induced by CREB during calorie restriction and may, in turn, increase CREB expression (and function) through a miRNA-mediated mechanism [33]. Consistently, recent evidence obtained in PC12 cell cultures indicates that the induction of CREB expression by IGF-I is mediated by downregulation of the microRNA miR-181a [34].

Taken together, the above evidence suggests that the complex interplay between SIRT1 and CREB, while affecting nutrient sensing and glucose homeostasis in peripheral tissues, may also play a pivotal role in the metabolic regulation of neuronal plasticity and of high-order brain functions.

## 3. Behavioral Outcomes and Effects on Learning and Memory

Hippocampal CA1 neurons display increased expression of the glucose transporter GLUT1 during the execution of a behavioral test [35]. This is an expected homeostatic reaction, aimed at fulfilling the increased metabolic demand of neurons challenged by a cognitive task. On the other hand, the finding that the molecular network outlined above (see Section 2) can actually* modulate* performance in behavioral tests involving learning and memory is less trivial.

Indeed, Zucker rats display impaired insulin sensitivity, which correlates with poor performance in the Morris Water Maze (MWM) [36]. In agreement with this finding, heterozygous knockout mice for insulin receptor show lower performance in the novel object recognition test [37]. Interestingly, lower values for glycosylated hemoglobin (HbA1c) and glycemia indicate improved glucose homeostasis and are associated with better performance on memory tasks in human subjects [38].

Moreover,* db/db* transgenic mice, which are a knockout for the gene encoding the leptin receptor, are also characterized by insulin-resistant diabetes [39]. Improving glucose homeostasis of* db/db* mice by means of physical exercise or calorie restriction ameliorates their exploratory behavior in an open field test [40]. It is noteworthy that this improvement is accompanied by increased expression of the* Bdnf* gene [40], possibly as a result of restored insulin signaling. Analogous results come from studies on the UCD-T2D mouse model of type 2 diabetes, which displays reduced hippocampal insulin signaling and reduced activation of the BDNF receptor, TrkB [41].

Moreover, knockout mice for the insulin receptor substrate p53 (IRSp53) display impaired learning and acquisition of a navigation task (MWM) and poor recognition memory (novel object recognition test, NOR) [42]. On the other hand, insulin receptor substrate 2 (IRS2) forebrain-specific knockout mice exhibit improved memory retention in the MWM test, whereas the learning curve is unaffected [43], indicating that different effectors of insulin can exert opposite actions on behavior.

Glucose intolerance can also result from exposure to a high-fat diet (HFD) during early postnatal life and is associated with impaired learning of an operant conditioning task (pressing a lever to obtain reward) and in the radial arm maze task [44]. Strikingly, this hippocampal-dependent behavioural task is unaffected if mice are subjected to HFD starting from adulthood [44]. It is tempting to speculate about the existence of a specific critical period(s) for developmental programming of proper sensitivity of hippocampal circuits to the various components of glucose signaling, in close analogy to what has been demonstrated for programming by environmental stimuli of the set point for hypothalamic leptin sensitivity [45] and the development of cortical sensory systems in response to early experience [46].

Similar data have been obtained from liver-specific, IGF-I knockout mice. They exhibit deficits in both the learning and memory retention phases of the MWM, which can be detected as early as two months of age [47] and still persist at 18 months of age [48]. In addition, treatment of young rats with an IGF-I antiserum impairs learning of a passive avoidance task [49].

These findings globally indicate that loss of insulin signaling results in decreased cognitive performance; conversely, administration of ghrelin and GLP-1 has been shown to improve learning and memory of new tasks.

Indeed, administration of ghrelin after training in a T-maze foot-shock avoidance test improves memory retention and, conversely, ghrelin knockout mice show impaired performance in the NOR test [50]. Consistently, bilateral intrahippocampal infusion of ghrelin for 4 days, prior to training in the MWM, enhances acquisition and memory retention of the task. Interestingly, this effect is abolished by coadministration of the PI3K antagonist LY294002 [51].

Lastly, GLP-1 receptor knockout mice display decreased memory retention in both the NOR and the MWM tests [52]. Conversely, intrahippocampal administration of GLP-1 to wild-type mice enhances spatial learning in both the passive avoidance and the MWM tests [19]. Moreover, administration of exendin-4, a GLP-1R agonist, for two weeks prior to training in a radial arm maze task is associated with improved spatial reference memory [53].

Taken together, these findings lend support to the view that molecules involved in glucose signaling play a key role in modulating learning and memory, with the intriguing implication that they can be exploited to potentiate cognitive function and to ameliorate pathological deficits.

## 4. Impact on Hippocampal Neurogenesis

The hippocampus is one of the few areas where neurogenesis persists throughout adulthood, thus supporting learning and memory, in addition to potentially contributing to brain repair [54]. In the adult mammalian brain, the subventricular and subgranular zones represent the two hippocampal neurogenic niches, populated by NSCs that proliferate and differentiate to generate new neurons [55]. A proper balance between the proliferative expansion of these populations and their maturation underlies the maintenance of both the hippocampus “stemness” reservoir and cognitive function [56, 57]. Although the regenerative potential of stem cell niches in the brain is still debated, a growing body of evidence indicates that, in the hippocampus, newborn neurons integrate into existing circuits to play a pivotal role in learning, memory, and neurological disorders [58].

Studies carried out both* in vitro* and* in vivo* suggest that insulin and IGF-I promote neurogenesis by affecting NSC proliferation, differentiation, and survival [59–61]. Moreover, insulin is a crucial trophic factor for nervous system development and maintenance of neurogenic niches. Indeed, activation of the insulin/IGF-I pathway is required for neuroblasts to exit quiescence [62, 63]. However, a chronic hyperstimulation of insulin/IGF-I effectors can lead to premature impoverishment of the NSC pool [64]. Therefore, insulin may exert either trophic or harmful effects on NSCs depending on the timing and the duration of stimulation.

On the other hand, animals undergoing calorie restriction exhibit lower plasma levels of glucose and insulin, in parallel with increased neurogenesis in the dentate gyrus [65] and slowdown of the age-related stemness decline [66]. Induction of the expression of the* Bdnf* gene [67] may at least partly explain the trophic action of nutrient deprivation on the NSC compartment. In addition, nutrient depletion may directly preserve the NSC capacity to self-renew and differentiate. In this regard, SIRT1 works as an epigenetic repressor and it modulates the neurogenic potential of neural precursors in the adult mouse brain niches [68]. According to an interesting scenario emerging from various experimental models, under metabolic and oxidative stress SIRT1 represses NSC self-renewal [69] and promotes their differentiation [70]. In summary, SIRT1 might serve as a metabolic sensor regulating the balance between NSC self-renewal and differentiation and controlling the preservation of the stem cell niche. Conversely, knocking out the genes encoding the nutrient- and insulin-regulated FoxO transcription factors causes sustained activation of nutrient replenishment signaling, thus leading to unbalanced proliferation and rapid exhaustion of neural progenitors both* in vivo* and* in vitro* [71]. Hence, absence of FoxOs mimics insulin hyperstimulation and promotes a premature senescence of the stem cell niche. Similarly, stimulation of the nutrient-dependent mTOR pathway causes reduced self-renewal and earlier NSC differentiation, resulting in altered brain development [72]. Accordingly, GLP-1 receptor agonist exendin-4 stimulates neurogenesis in the dentate gyrus, evaluated by bromodeoxyuridine incorporation assay, as well as by the expression of the newborn neuron marker doublecortin [53]. Moreover, the antidiabetic drug sitagliptin, in concomitance with the amelioration of peripheral glucose homeostasis, improves hippocampal neurogenesis and recognition memory through the upregulation of hippocampal GLP-1 receptor, in addition to modifying the expression of key genes involved in cognitive decline [73].

Together, this evidence supports the idea that nutrient-related signals control NSC fate, actively participating in neural plasticity processes, under both physiological and pathological (i.e., overnutrition, diabetes and see Section 6) conditions.

## 5. Effects on Synaptogenesis and Synaptic Plasticity

Modifications in the activity of synapses, that is, potentiation or depression, or in their function and number, for example, generation of new dendritic spines, represent the functional and structural substrates underlying the integration of neurons into networks. The interaction between these different phenomena is, in turn, instrumental to acquire and consolidate behavioral modifications (see Section 3).

Treatment of primary cultures of rat hippocampal neurons results in higher frequency of miniature excitatory postsynaptic currents (mEPSCs), suggesting an increased basal neurotransmitter release from presynaptic terminals [74]. This functional effect is paralleled by increased density of dendritic spines, involving activation of the Akt pathway and of the Rho GTPase Rac1, an important mediator of cytoskeleton rearrangement [74].

However, the most interesting effects are observed on activity-dependent synaptic plasticity. First, Zucker rats display loss of insulin sensitivity and a concurrent reduction in long-term potentiation (LTP) at CA3–CA1 synapses, whereas long-term depression (LTD) is unaffected [36]. In addition, heterozygous knockout mice for insulin receptor have normal basal synaptic transmission and induction of LTP that, however, fails to be consolidated owing to reduced activation of the Akt pathway [37].

On the other hand, in physiological conditions insulin facilitates LTP at hippocampal synapses. Van der Heide and colleagues [75] have shown that insulin application results in a leftward shift in the input-output relationship of excitatory postsynaptic potentials (EPSPs) response as a function of stimulation frequency. Indeed, under control conditions, LTD or LTP can be achieved using stimulation frequencies of 1 Hz and 50–100 Hz, respectively. However, in the presence of insulin LTD or LTP is obtained in response to stimulation frequencies of 0.033 Hz or 10 Hz (which would yield no effect under control conditions), respectively [75]. This can be interpreted as a metaplastic effect, that is, to a “plasticity of plasticity” phenomenon that results in a lower stimulation frequency threshold, called “*θ*_*m*_,” required for obtaining LTP [76]. This reinforces the view that insulin is a modulator rather than an “inducer” of synaptic plasticity.

In keeping with the findings shown in Section 2, this effect requires activation of the PI3K pathway [75] and results in increased exocytosis of N-methyl-D-aspartate receptors (NMDARs) [77]. Moreover, NMDAR function is also transiently enhanced by phosphorylation of the NR2A and NR2B subunits [78], which correlates with the potentiation of NMDAR-mediated currents [79]. It is worth noting that NR2A and NR2B subunits are responsible for different NMDA current kinetics [80]. In addition, NR2B confers a higher time constant to NMDA responses, predominates during early cortical and hippocampal development, and is downregulated in adulthood, when NR2A becomes more expressed [81].

Insulin treatment of hippocampal cultures also increases phosphorylation of the GluR1 subunit of *α*-amino-3-hydroxy-5-methyl-4-isoxazolepropionic acid receptors (AMPARs) [82], indicating that multiple sites of action are responsible for the effect of insulin on synaptic plasticity.

Finally, in thalamocortical organotypic slices this hormone stimulates maturation of silent synapses, that is, those mainly containing NMDARs that represent a substrate for circuit potentiation through AMPARs insertion [83].

Notably, these data were obtained following acute insulin applications, whereas chronic elevation of cerebral insulin levels by means of intracerebroventricular infusion greatly reduces LTP in the CA1 area in response to high-frequency stimulation [84]. However, the mechanisms responsible for this time-dependent change in the polarity of insulin effect on synaptic plasticity still need to be better addressed.

Insulin receptor substrates are essential to the actuation of the above described effects. For instance, the synapse-specific IRSp53 [85] interacts with the activated Rho GTPase Rac [86] and with the postsynaptic protein PSD-95 [87]. Accordingly, overexpression of IRSp53 stimulates dendritic spine formation [87]. However, transgenic mice lacking the gene encoding this protein show enhanced LTP of the Schaffer collateral pathway, although they do not display any obvious change in dendritic spine density and morphology [42]. The higher propensity of IRSp53 knockout mice for LTP correlates with increased NMDAR-dependent synaptic transmission, although no obvious changes in the expression of NMDAR and AMPAR subunits could be detected [42]. It is noteworthy that another group independently generated an* IRSp53* ^−/−^ transgenic mouse line and found a small (17%), but significant, reduction in postsynaptic density area, in addition to the upregulation in the expression of NR2A and NR2B proteins in both juvenile and adult animals [88]. Taken together, these data point to a role of IRSp53 in promoting generation of dendritic spines which, on the other hand, are less sensitive to display LTP, although the underlying mechanisms are still unclear.

Interestingly, also IRS2 deletion affects structural and functional plasticity of the hippocampus, but with different outcomes in comparison to IRSp53. Indeed, IRS2 knockout mice have higher density of CA1 dendritic spines [43], in addition to showing decreased LTP at the Schaffer collateral pathway, as a result of impaired Akt activation and lower phosphorylation of NR2B subunits [89].

It is important to notice that the morphology of dendritic spines (e.g., unstable filopodia versus stable mushroom spines) was not assessed in these studies, and elucidation of this issue would contribute to understanding the seemingly contrasting effects of manipulating the expression of different IRSs on structural and functional plasticity.

Considering the convergence on the same molecular effectors as insulin, it is not surprising that IGF-1 treatment stimulates structural plasticity in cortical cultures, as assessed by increased immunoreactivity for synaptic markers such as synapsin-1 and PSD-95 [90]. In addition, IGF-I knockout mice have reduced dendritic complexity and number of dendritic spines of cortical layer II-III neurons [91]. This can represent one of the substrates for the role of IGF-1 in promoting plasticity. A similar action is likely exerted in the adult hippocampus, since administration of IGF-I antiserum partially blocks the increase in spine density of CA1 basal dendrites in response to physical exercise [92]. Moreover, liver-specific IGF-I knockout mice exhibit impaired LTP at perforant path-dentate gyrus synapses. This deficit is partially rescued if the inhibitory tone is decreased by bath perfusion with the GABA_A_ receptor antagonist bicuculline. Consistently, IGF-I knockout mice have reduced density of glutamatergic synapses, which leads to a lower excitation/inhibition ratio [47].

During development, brain-specific overexpression of human IGF-I results in boosting of postnatal synaptogenesis in the molecular layer of the dentate gyrus peaking at 35 days of age [93]. This phenomenon is likely the result of accelerated maturation, since the final number of synapses is not different from that of wild-type controls [93].

Further investigation is required to understand whether IGF-I overexpression has any consequence on the developmental curve of hippocampal neurons at functional, structural, and behavioral levels. Besides, the therapeutic potential of IGF-1 in diseases characterized by impaired hippocampal function needs to be better investigated, especially considering evidence pointing to brain insulin and IGF-1 resistance in Alzheimer's disease patients [94].

Analogous effects are exerted by ghrelin, which crosses the blood-brain barrier to bind its hippocampal receptors. Indeed, peripheral administration of ghrelin results in higher density of dendritic spines in the CA1 area and in augmentation of LTP of the Schaffer collateral pathway [50]. Moreover, addition of ghrelin to rat hippocampal slices increases the density of phalloidin-positive puncta, which indicates higher abundance of polymerized F-actin, thus representing an indirect measurement of dendritic spine reorganization [95]. Although more accurate measurements need to be performed, for instance, with the use of time-lapse imaging on slices from GFP-expressing transgenic mice [96], this finding is suggestive of increased dendritic spine dynamics.

Recent data indicate that GHS-R1as are expressed in the vicinity of hippocampal excitatory synapses and, indeed, their pharmacological activation triggers surface exposure of GluA1 subunits of the AMPA glutamate receptor [24]. This structural change is paralleled by facilitation of NMDA receptor-dependent LTP via PI3K/Akt activation [24]. In addition, ghrelin also stimulates phosphorylation of the NR1 subunit of NMDARs, which can further facilitate activity-dependent synaptic plasticity [22]. Moreover, experiments on rat brain slices containing substantia nigra pars compacta have shown that ghrelin enhances excitability also by inhibiting Kv7 channels [97]. It would be interesting to study whether a similar mechanism is also present in the hippocampus.

Finally, GLP-1 appears to affect mainly basal inhibitory synaptic transmission, as it has been shown to increase both frequency and amplitude of GABAergic spontaneous inhibitory postsynaptic currents (sIPSCs) recorded from CA3 neurons [98]. Accordingly, GLP-1 reduces excitability of hippocampal cultures by acting on glutamate- and depolarization-induced Ca^2+^ influx; this effect has been hypothesized to have the purpose of protecting neurons from glutamate excitotoxicity [99], as it occurs, for instance, in epilepsy. Indeed, GLP-1 receptor knockout mice have lower threshold for and higher severity of kainic acid-induced seizures [19]. However, it should be taken into account that neuronal response to GLP-1 can vary according to the time-scale considered. As it has been shown for CA1 neurons by Oka and colleagues using* in vivo* electrophysiological recordings in anesthetized rats, an initial increase in single-unit activity is followed by a decrease [100]. Moreover, the effect of GLP-1 on synaptic plasticity appears to be radically different from that on basal transmission. Indeed, administration of GLP-1 receptor agonists such as liraglutide increases LTP [73], whereas GLP-1 receptor knockout mice show impairment in this form of synaptic plasticity [52].

Hence, the data summarized above indicate that structural and functional aspects of hippocampal plasticity are strongly sensitive to key mediators of glucose homeostasis. Moreover, they suggest the existence of multiple interactions and synergies between the different molecular players, and understanding the details of this network appears to be one of the main goals of future research.

## 6. Effects of Glucose Homeostasis Dysregulation on Hippocampal Plasticity

The data reviewed in the previous paragraphs support the view that glucose homeostasis imbalances can alter signaling pathways involved in adult neurogenesis and synaptic plasticity, thereby leading to reduced “mindspan” (the maintenance of mental abilities throughout life) and increased risk of neurodegenerative disorders [101]. Moreover, it is widely known that energy restriction promotes neuronal survival and improves cognitive function [6]. Conversely, the excess of nutrients impinges on brain health and impairs synaptic transmission and plasticity leading to accelerated cognitive decline (CD) [102, 103].

In line with these concepts, humans in the Western world are thought to be “unnaturally” overfed and sedentary, a state of chronic positive energy balance that results in suboptimal health [104]. In addition, the incidence of metabolic disorders, including type 2 diabetes (T2D), is increasing at alarming rates worldwide, largely due to poor lifestyle habits. In parallel, the prevalence of CD also increases as the world population ages [105].

Epidemiological/clinical observations have accumulated showing that diabetic patients are significantly more likely to develop cognitive impairment and exhibit increased susceptibility to dementia. Importantly, impaired metabolic parameters, such as hyperglycemia and hyperinsulinemia, positively correlate with CD [106]. Elevated blood glucose levels increase the risk of dementia in both diabetic and nondiabetic individuals by 40% and 18%, respectively, [107] and are associated with CD and reduced hippocampal volume [38]. These findings indicate that fluctuations in blood glucose levels negatively impact on brain function, even in the absence of overt T2D or impaired glucose tolerance.

Chronic hyperglycemia and hyperinsulinemia primarily stimulate the formation of advanced glucose end products, which leads to an overproduction of reactive oxygen species and alteration of intracellular second messenger pathways [108]. Whereas insulin is clearly neurotrophic at moderate concentrations, too much insulin in the brain may be associated with increased amyloid-*β* deposition due to competition for their common and main clearance mechanism, the insulin-degrading enzyme [109]. In this regard, it has even been proposed that Alzheimer's disease may be considered a form of type 3 diabetes, based on the evidence for insulin resistance and impaired insulin-response pathways in the Alzheimer's-affected brain [110]. However, glucose and insulin levels changes are not the only metabolic factors involved in hippocampal plasticity alterations produced by glucose dyshomeostasis.

Interestingly, in insulin-deficient rats and insulin-resistant mice, diabetes impairs hippocampus-dependent memory, impinging on both synaptic plasticity and adult neurogenesis, and the glucocorticoid system contributes to these adverse effects [111]. In this regard, NSC proliferation and adult neurogenesis are impaired in T2D and prediabetes animal models [112].

Moreover, as mentioned above, several gut hormones are able to impact on hippocampal function. An additional aspect to be taken into account is that microbiota dysbiosis could affect the gut-brain axis, thus promoting insulin resistance and cognitive impairment [113]. In addition, germ-free mice show a significant alteration of serotonergic system metabolites concentration and serotonergic neurotransmission in the hippocampus [114], which can have a negative impact on synaptic plasticity.

Hence, a key topic in current research is understanding which metabolic factors are most harmful to brain plasticity and which drugs suitable for metabolic disorders can also have an effect on cognitive functions. In particular, a challenge for the upcoming years will be investigating whether there are common molecular mechanisms underlying metabolic and neurodegenerative diseases and whether the “glycemic memory” of particular brain areas (e.g., the hippocampus) may be a risk factor for early CD.

## 7. Concluding Remarks and Future Perspectives

Molecules involved in metabolic homeostasis are now recognized to exert a deep influence on hippocampal plasticity and alteration of their equilibrium has a strong impact at the functional and behavioral levels.

It is worth noting that, for instance, experimental paradigms such as physical exercise or environmental enrichment, that is, coupling of motor activity to sensory stimulation, social interaction, and enhancement of exploratory behavior [115], dramatically affect neural plasticity of several brain districts, including the hippocampus, during development and in adulthood, as well as in aging [115–117]. Interestingly, this effect is correlated to enhanced glucose tolerance [45]. Therefore, metabolically active molecules can act as a bridge between a healthy body and a healthy brain by communicating a status of optimal metabolic homeostasis, which can represent a modulatory (or permissive) factor for the activation of neuroplasticity, that is, a highly energy-demanding process. In this regard, understanding how brain sensitivity to insulin and other metabolic players can be controlled may be an effective way to impact on pathologies characterized by impaired neural plasticity, especially Alzheimer's disease. Indeed, expression of the insulin receptor mRNA is maximal during development and decreases with aging [118], and this, in addition to representing another similarity between insulin sensitivities inside and outside the brain, could represent a key contributor to the decline in neural plasticity of the elderly. Thus, acting on insulin signaling might be a promising strategy for overcoming age-associated plasticity deficits.

Finally, a crucial point is also represented by understanding interactions between glucose and lipid homeostasis, since soluble factors involved in this latter process, such as leptin, have also been demonstrated to modulate synaptic plasticity and to have a role in age-associated CD [45, 119, 120].

In summary, drawing a comprehensive picture of the interactions between metabolism, hippocampal circuits, and cognitive performance, in addition to elucidating the underlying molecular mechanisms, can represent an important step forward, from a conceptual and translational point of view, towards a deeper understanding of the mechanisms regulating neural plasticity in health and neurodegeneration.

## References

[1] Verberne A. J. M., Sabetghadam A., Korim W. S. (2014). Neural pathways that control the glucose counterregulatory response. *Frontiers in Neuroscience*.

[2] Williams K. W., Elmquist J. K. (2012). From neuroanatomy to behavior: central integration of peripheral signals regulating feeding behavior. *Nature Neuroscience*.

[3] Jordan S. D., Könner A. C., Brüning J. C. (2010). Sensing the fuels: glucose and lipid signaling in the CNS controlling energy homeostasis. *Cellular and Molecular Life Sciences*.

[4] Folli F., Ghidella S., Bonfanti L., Kahn C. R., Merighi A. (1996). The early intracellular signaling pathway for the insulin/insulin-like growth factor receptor family in the mammalian central nervous system. *Molecular Neurobiology*.

[5] Hwang J.-I., Yun S., Moon M. J., Park C. R., Seong J. Y. (2014). MOlecular evolution of GPCRs: GLP1/GLP1 receptors. *Journal of Molecular Endocrinology*.

[6] Fusco S., Pani G. (2013). Brain response to calorie restriction. *Cellular and Molecular Life Sciences*.

[7] Howarth C., Gleeson P., Attwell D. (2012). Updated energy budgets for neural computation in the neocortex and cerebellum. *Journal of Cerebral Blood Flow and Metabolism*.

[8] Ivannikov M. V., Sugimori M., Llinás R. R. (2010). Calcium clearance and its energy requirements in cerebellar neurons. *Cell Calcium*.

[9] Dienel G. A. (2012). Fueling and imaging brain activation. *ASN Neuro*.

[10] Kim D. Y., Rhee I., Paik J. (2014). Metabolic circuits in neural stem cells. *Cellular and Molecular Life Sciences*.

[11] Hertz L., Gibbs M. E. (2009). What learning in day-old chickens can teach a neurochemist: focus on astrocyte metabolism. *Journal of Neurochemistry*.

[12] Suzuki A., Stern S. A., Bozdagi O. (2011). Astrocyte-neuron lactate transport is required for long-term memory formation. *Cell*.

[13] Simpson I. A., Carruthers A., Vannucci S. J. (2007). Supply and demand in cerebral energy metabolism: the role of nutrient transporters. *Journal of Cerebral Blood Flow and Metabolism*.

[14] Blazquez E., Velazquez E., Hurtado-Carneiro V. (2014). Insulin in the brain: its pathophysiological implications for States related with central insulin resistance, type 2 diabetes and Alzheimer's disease. *Frontiers in Endocrinology (Lausanne)*.

[15] Fernandez A. M., Torres-Alemán I. (2012). The many faces of insulin-like peptide signalling in the brain. *Nature Reviews Neuroscience*.

[16] Holst J. J. (2007). The physiology of glucagon-like peptide 1. *Physiological Reviews*.

[17] Hölscher C., Hamilton A. (2009). Receptors for the incretin glucagon-like peptide-1 are expressed on neurons in the central nervous system. *NeuroReport*.

[18] Merchenthaler I., Lane M., Shughrue P. (1999). Distribution of pre-pro-glucagon and glucagon-like peptide-1 receptor messenger RNAs in the rat central nervous system. *Journal of Comparative Neurology*.

[19] During M. J., Cao L., Zuzga D. S. (2003). Glucagon-like peptide-1 receptor is involved in learning and neuroprotection. *Nature Medicine*.

[20] Andrews Z. B., Abizaid A. (2014). Neuroendocrine mechanisms that connect feeding behavior and stress. *Frontiers in Neuroscience*.

[21] Broglio F., Arvat E., Benso A. (2001). Ghrelin, a natural gh secretagogue produced by the stomach, induces hyperglycemia and reduces insulin secretion in humans. *Journal of Clinical Endocrinology and Metabolism*.

[22] Cuellar J. N., Isokawa M. (2011). Ghrelin-induced activation of cAMP signal transduction and its negative regulation by endocannabinoids in the hippocampus. *Neuropharmacology*.

[23] Frago L. M., E. Baquedano,, Argente J., Chowen  J. A. (2011). Neuroprotective actions of ghrelin and growth hormone secretagogues. *Frontiers in Molecular Neuroscience*.

[24] Ribeiro L. F., Catarino T., Santos S. D. (2014). Ghrelin triggers the synaptic incorporation of AMPA receptors in the hippocampus. *Proceedings of the National Academy of Sciences of the United States of America*.

[25] Reichardt L. F. (2006). Neurotrophin-regulated signalling pathways. *Philosophical Transactions of the Royal Society B: Biological Sciences*.

[26] Altarejos J. Y., Montminy M. (2011). CREB and the CRTC co-activators: sensors for hormonal and metabolic signals. *Nature Reviews Molecular Cell Biology*.

[27] Finkbeiner S. (2000). CREB couples neurotrophin signals to survival messages. *Neuron*.

[28] Fusco S., Maulucci G., Pani G. (2012). Sirt1: def-eating senescence?. *Cell Cycle*.

[29] Du L.-L., Xie J.-Z., Cheng X.-S. (2014). Activation of sirtuin 1 attenuates cerebral ventricular streptozotocin-induced tau hyperphosphorylation and cognitive injuries in rat hippocampi. *Age*.

[30] Jeong H., Cohen D. E., Cui L. (2012). Sirt1 mediates neuroprotection from mutant huntingtin by activation of the TORC1 and CREB transcriptional pathway. *Nature Medicine*.

[31] Fusco S., Ripoli C., Podda M. V. (2012). A role for neuronal cAMP responsive-element binding (CREB)-1 in brain responses to calorie restriction. *Proceedings of the National Academy of Sciences of the United States of America*.

[32] Cohen D. E., Supinski A. M., Bonkowski M. S. (2009). Neuronal SIRT1 regulates endocrine and behavioral responses to calorie restriction. *Genes & Development*.

[33] Gao J., Wang W.-Y., Mao Y.-W. (2010). A novel pathway regulates memory and plasticity via SIRT1 and miR-134. *Nature*.

[34] Liu Y., Zhao Z., Yang F., Gao Y., Song J., Wan Y. (2013). microRNA-181a is involved in insulin-like growth factor-1-mediated regulation of the transcription factor CREB1. *Journal of Neurochemistry*.

[35] Choeiri C., Staines W., Miki T., Seino S., Messier C. (2005). Glucose transporter plasticity during memory processing. *Neuroscience*.

[36] Kamal A., Ramakers G. M. J., Gispen W. H., Biessels G. J., Al Ansari A. (2013). Hyperinsulinemia in rats causes impairment of spatial memory and learning with defects in hippocampal synaptic plasticity by involvement of postsynaptic mechanisms. *Experimental Brain Research*.

[37] Nisticó R., Cavallucci V., Piccinin S. (2012). Insulin receptor *β*-subunit haploinsufficiency impairs hippocampal late-phase ltp and recognition memory. *NeuroMolecular Medicine*.

[38] Kerti L., Witte A. V., Winkler A., Grittner U., Rujescu D., Flöel A. (2013). Higher glucose levels associated with lower memory and reduced hippocampal microstructure. *Neurology*.

[39] Chen H., Charlat O., Tartaglia L. A. (1996). Evidence that the diabetes gene encodes the leptin receptor: identification of a mutation in the leptin receptor gene in db/db mice. *Cell*.

[40] Stranahan A. M., Lee K., Martin B. (2009). Voluntary exercise and caloric restriction enhance hippocampal dendritic spine density and BDNF levels in diabetic mice. *Hippocampus*.

[41] Agrawal R., Zhuang Y., Cummings B. P. (2014). Deterioration of plasticity and metabolic homeostasis in the brain of the UCD-T2DM rat model of naturally occurring type-2 diabetes. *Biochimica et Biophysica Acta—Molecular Basis of Disease*.

[42] Kim M.-H., Choi J., Yang J. (2009). Enhanced NMDA receptor-mediated synaptic transmission, enhanced long-term potentiation, and impaired learning and memory in mice lacking IRSp53. *Journal of Neuroscience*.

[43] Irvine E. E., Drinkwater L., Radwanska K. (2011). Insulin receptor substrate 2 is a negative regulator of memory formation. *Learning & Memory*.

[44] Boitard C., Etchamendy N., Sauvant J. (2012). Juvenile, but not adult exposure to high-fat diet impairs relational memory and hippocampal neurogenesis in mice. *Hippocampus*.

[45] Mainardi M., Scabia G., Vottari T. (2010). A sensitive period for environmental regulation of eating behavior and leptin sensitivity. *Proceedings of the National Academy of Sciences of the United States of America*.

[46] Berardi N., Pizzorusso T., Maffei L. (2000). Critical periods during sensory development. *Current Opinion in Neurobiology*.

[47] Trejo J. I., Piriz J., Llorens-Martin M. V. (2007). Central actions of liver-derived insulin-like growth factor I underlying its pro-cognitive effects. *Molecular Psychiatry*.

[48] Svensson J., Diez M., Engel J. (2006). Endocrine, liver-derived IGF-I is of importance for spatial learning and memory in old mice. *Journal of Endocrinology*.

[49] Lupien S. B., Bluhm E. J., Ishii D. N. (2003). Systemic insulin-like growth factor-I administration prevents cognitive impairment in diabetic rats, and brain IGF regulates learning/memory in normal adult rats. *Journal of Neuroscience Research*.

[50] Diano S., Farr S. A., Benoit S. C. (2006). Ghrelin controls hippocampal spine synapse density and memory performance. *Nature Neuroscience*.

[51] Chen L., Xing T., Wang M. (2011). Local infusion of ghrelin enhanced hippocampal synaptic plasticity and spatial memory through activation of phosphoinositide 3-kinase in the dentate gyrus of adult rats. *European Journal of Neuroscience*.

[52] Abbas T., Faivre E., Hölscher C. (2009). Impairment of synaptic plasticity and memory formation in GLP-1 receptor KO mice: interaction between type 2 diabetes and Alzheimer's disease. *Behavioural Brain Research*.

[53] Isacson R., Nielsen E., Dannaeus K. (2011). The glucagon-like peptide 1 receptor agonist exendin-4 improves reference memory performance and decreases immobility in the forced swim test. *European Journal of Pharmacology*.

[54] Braun S. M. G., Jessberger S. (2014). Review: adult neurogenesis and its role in neuropsychiatric disease, brain repair and normal brain function. *Neuropathology and Applied Neurobiology*.

[55] Urbán N., Guillemot F. (2014). Neurogenesis in the embryonic and adult brain: same regulators, different roles. *Frontiers in Cellular Neuroscience*.

[56] Castilla-Ortega E., Pedraza C., Estivill-Torrús G., Santín L. J. (2011). When is adult hippocampal neurogenesis necessary for learning? Evidence from animal research. *Reviews in the Neurosciences*.

[57] Lee S. W., Clemenson G. D., Gage F. H. (2012). New neurons in an aged brain. *Behavioural Brain Research*.

[58] Taylor C. J., Jhaveri D. J., Bartlett P. F. (2013). The therapeutic potential of endogenous hippocampal stem cells for the treatment of neurological disorders. *Frontiers in Cellular Neuroscience*.

[59] Åberg M. A. I., Åberg N. D., Palmer T. D. (2003). IGF-I has a direct proliferative effect in adult hippocampal progenitor cells. *Molecular and Cellular Neuroscience*.

[60] Brooker G. J. F., Kalloniatis M., Russo V. C., Murphy M., Werther G. A., Bartlett P. F. (2000). Endogenous IGF-1 regulates the neuronal differentiation of adult stem cells. *Journal of Neuroscience Research*.

[61] Drago J., Murphy M., Carroll S. M., Harvey R. P., Bartlett P. F. (1991). Fibroblast growth factor-mediated proliferation of central nervous system precursors depends on endogenous production of insulin-like growth factor I. *Proceedings of the National Academy of Sciences of the United States of America*.

[62] Chell J. M., Brand A. H. (2010). Nutrition-responsive glia control exit of neural stem cells from quiescence. *Cell*.

[63] Sousa-Nunes R., Yee L. L., Gould A. P. (2011). Fat cells reactivate quiescent neuroblasts via TOR and glial insulin relays in *Drosophila*. *Nature*.

[64] Sun L. Y. (2006). Hippocampal IGF-1 expression, neurogenesis and slowed aging: clues to longevity from mutant mice. *Age*.

[65] Lee J., Seroogy K. B., Mattson M. P. (2002). Dietary restriction enhances neurotrophin expression and neurogenesis in the hippocampus of adult mice. *Journal of Neurochemistry*.

[66] Dash P. K., Mach S. A., Moore A. N. (2001). Enhanced neurogenesis in the rodent hippocampus following traumatic brain injury. *Journal of Neuroscience Research*.

[67] Maswood N., Young J., Tilmont E. (2004). Caloric restriction increases neurotrophic factor levels and attenuates neurochemical and behavioral deficits in a primate model of Parkinson's disease. *Proceedings of the National Academy of Sciences of the United States of America*.

[68] Saharan S., Jhaveri D. J., Bartlett P. F. (2013). SIRT1 regulates the neurogenic potential of neural precursors in the adult subventricular zone and hippocampus. *Journal of Neuroscience Research*.

[69] Ma C., Yao M., Zhai Q., Jiao J., Yuan X., Poo M. (2014). SIRT1 suppresses self-renewal of adult hippocampal neural stem cells. *Development*.

[70] Prozorovski T., Schulze-Topphoff U., Glumm R. (2008). Sirt1 contributes critically to the redox-dependent fate of neural progenitors. *Nature Cell Biology*.

[71] Renault V. M., Rafalski V. A., Morgan A. A. (2009). FoxO3 regulates neural stem cell homeostasis. *Cell Stem Cell*.

[72] Magri L., Cambiaghi M., Cominelli M. (2011). Sustained activation of mTOR pathway in embryonic neural stem cells leads to development of tuberous sclerosis complex-associated lesions. *Cell Stem Cell*.

[73] Gault V. A., Hölscher C. (2008). GLP-1 agonists facilitate hippocampal LTP and reverse the impairment of LTP induced by beta-amyloid. *European Journal of Pharmacology*.

[74] Lee C.-C., Huang C.-C., Hsu K.-S. (2011). Insulin promotes dendritic spine and synapse formation by the PI3K/Akt/mTOR and Rac1 signaling pathways. *Neuropharmacology*.

[75] van der Heide L. P., Kamal A., Artola A., Gispen W. H., Ramakers G. M. J. (2005). Insulin modulates hippocampal activity-dependent synaptic plasticity in a N-methyl-D-aspartate receptor and phosphatidyl-inositol-3-kinase-dependent manner. *Journal of Neurochemistry*.

[76] Abraham W. C. (2008). Metaplasticity: tuning synapses and networks for plasticity. *Nature Reviews Neuroscience*.

[77] Skeberdis V. A., Lan J.-Y., Zheng X., Zukin R. S., Bennett M. V. L. (2001). Insulin promotes rapid delivery of *N*-methyl-d-aspartate receptors to the cell surface by exocytosis. *Proceedings of the National Academy of Sciences of the United States of America*.

[78] Christie J. M., Wenthold R. J., Monaghan D. T. (1999). Insulin causes a transient tyrosine phosphorylation of NR2A and NR2B NMDA receptor subunits in rat hippocampus. *Journal of Neurochemistry*.

[79] Liu L., Brown J. C., Webster W. W., Morrisett R. A., Monaghan D. T. (1995). Insulin potentiates N-methyl-d-aspartate receptor activity in *Xenopus* oocytes and rat hippocampus. *Neuroscience Letters*.

[80] Barria A., Malinow R. (2005). NMDA receptor subunit composition controls synaptic plasticity by regulating binding to CaMKII. *Neuron*.

[81] Gambrill A. C., Barria A. (2011). NMDA receptor subunit composition controls synaptogenesis and synapse stabilization. *Proceedings of the National Academy of Sciences of the United States of America*.

[82] Adzovic L., Domenici L. (2014). Insulin induces phosphorylation of the AMPA receptor subunit GluR1, reversed by ZIP, and over-expression of Protein Kinase M zeta, reversed by amyloid beta. *Journal of Neurochemistry*.

[83] Plitzko D., Rumpel S., Gottmann K. (2001). Insulin promotes functional induction of silent synapses in differentiating rat neocortical neurons. *European Journal of Neuroscience*.

[84] Kamal A., Ramakers G. M. J., Gispen W. H., Biessels G. J. (2012). Effect of chronic intracerebroventricular insulin administration in rats on the peripheral glucose metabolism and synaptic plasticity of CA1 hippocampal neurons. *Brain Research*.

[85] Abbott M.-A., Wells D. G., Fallon J. R. (1999). The insulin receptor tyrosine kinase substrate p58/53 and the insulin receptor are components of CNS synapses. *The Journal of Neuroscience*.

[86] Miki H., Yamaguchi H., Suetsugu S., Takenawa T. (2000). IRSp53 is an essential intermediate between Rac and WAVE in the regulation of membrane ruffling. *Nature*.

[87] Choi J., Ko J., Racz B. (2005). Regulation of dendritic spine morphogenesis by insulin receptor substrate 53, a downstream effector of Rac1 and Cdc42 small GTPases. *Journal of Neuroscience*.

[88] Sawallisch C., Berhörster K., Disanza A. (2009). The insulin receptor substrate of 53 kDa (IRSp53) limits hippocampal synaptic plasticity. *The Journal of Biological Chemistry*.

[89] Martín E. D., Sánchez-Perez A., Trejo J. L. (2012). IRS-2 deficiency impairs NMDA receptor-dependent long-term potentiation. *Cerebral Cortex*.

[90] Corvin A. P., Molinos I., Little G. (2012). Insulin-like growth factor 1 (IGF1) and its active peptide (1–3)IGF1 enhance the expression of synaptic markers in neuronal circuits through different cellular mechanisms. *Neuroscience Letters*.

[91] Cheng C. M., Mervis R. F., Niu S.-L. (2003). Insulin-like growth factor 1 is essential for normal dendritic growth. *Journal of Neuroscience Research*.

[92] Glasper E. R., Llorens-Martin M. V., Leuner B., Gould E., Trejo J. L. (2010). Blockade of insulin-like growth factor-I has complex effects on structural plasticity in the hippocampus. *Hippocampus*.

[93] O’Kusky J. R., Ye P., D’Ercole A. J. (2000). Insulin-like growth factor-I promotes neurogenesis and synaptogenesis in the hippocampal dentate gyrus during postnatal development. *Journal of Neuroscience*.

[94] Talbot K., Wang H.-Y., Kazi H. (2012). Demonstrated brain insulin resistance in Alzheimer's disease patients is associated with IGF-1 resistance, IRS-1 dysregulation, and cognitive decline. *The Journal of Clinical Investigation*.

[95] Berrout L., Isokawa M. (2012). Ghrelin promotes reorganization of dendritic spines in cultured rat hippocampal slices. *Neuroscience Letters*.

[96] Mainen Z. F., Maletic-Savatic M., Shi S. H., Hayashi Y., Malinow R., Svoboda K. (1999). Two-photon imaging in living brain slices. *Methods*.

[97] Shi L., Bian X., Qu Z. (2013). Peptide hormone ghrelin enhances neuronal excitability by inhibition of Kv7/KCNQ channels. *Nature Communications*.

[98] Korol S. V., Jin Z., Babateen O., Birnir B. (2014). GLP-1 and exendin-4 transiently enhance GABAA receptor-mediated synaptic and tonic currents in rat hippocampal CA3 pyramidal neurons. *Diabetes*.

[99] Gilman C. P., Perry T. A., Furukawa K., Grieg N. H., Egan J. M., Mattson M. P. (2003). Glucagon-like peptide 1 modulates calcium responses to glutamate and membrane depolarization in hippocampal neurons. *Journal of Neurochemistry*.

[100] Oka J.-I., Goto N., Kameyama T. (1999). Glucagon-like peptide-1 modulates neuronal activity in the rat's hippocampus. *NeuroReport*.

[101] Kodl C. T., Seaquist E. R. (2008). Cognitive dysfunction and diabetes mellitus. *Endocrine Reviews*.

[102] Elias M. F., Goodell A. L., Waldstein S. R. (2012). Obesity, cognitive functioning and dementia: back to the future. *Journal of Alzheimer's Disease*.

[103] Sellbom K. S., Gunstad J. (2012). Cognitive function and decline in obesity. *Journal of Alzheimer's Disease*.

[104] Martin B., Ji S., Maudsley S., Mattson M. P. (2010). ‘Control’ laboratory rodents are metabolically morbid: why it matters. *Proceedings of the National Academy of Sciences of the United States of America*.

[105] Kirk-Sanchez N. J., McGough E. L. (2013). Physical exercise and cognitive performance in the elderly: current perspectives. *Clinical Interventions in Aging*.

[106] Cukierman-Yaffee T. (2009). The relationship between dysglycemia and cognitive dysfunction. *Current Opinion in Investigational Drugs*.

[107] Crane P. K., Walker R., Larson E. B. (2013). Glucose levels and risk of dementia. *The New England Journal of Medicine*.

[108] Brownlee M. (2001). Biochemistry and molecular cell biology of diabetic complications. *Nature*.

[109] Farris W., Mansourian S., Chang Y. (2003). Insulin-degrading enzyme regulates the levels of insulin, amyloid *β*-protein, and the *β*-amyloid precursor protein intracellular domain in vivo. *Proceedings of the National Academy of Sciences of the United States of America*.

[110] Steen E., Terry B. M., Rivera E. J. (2005). Impaired insulin and insulin-like growth factor expression and signaling mechanisms in Alzheimer's disease—is this type 3 diabetes?. *Journal of Alzheimer's Disease*.

[111] Stranahan A. M., Arumugam T. V., Cutler R. G., Lee K., Egan J. M., Mattson M. P. (2008). Diabetes impairs hippocampal function through glucocorticoid-mediated effects on new and mature neurons. *Nature Neuroscience*.

[112] Ramos-Rodriguez J. J., Molina-Gil S., Ortiz-Barajas O. (2014). Central proliferation and neurogenesis is impaired in type 2 diabetes and prediabetes animal models. *PLoS ONE*.

[113] Daulatzai M. A. (2014). Chronic functional bowel syndrome enhances gut-brain axis dysfunction, neuroinflammation, cognitive impairment, and vulnerability to dementia. *Neurochemical Research*.

[114] Clarke G., Grenham S., Scully P. (2013). The microbiome-gut-brain axis during early life regulates the hippocampal serotonergic system in a sex-dependent manner. *Molecular Psychiatry*.

[115] Sale A., Berardi N., Maffei L. (2014). Environment and brain plasticity: towards an endogenous pharmacotherapy. *Physiological Reviews*.

[116] Mainardi M., di Garbo A., Caleo M., Berardi N., Sale A., Maffei L. (2014). Environmental enrichment strengthens corticocortical interactions and reduces amyloid-*β* oligomers in aged mice. *Frontiers in Aging Neuroscience*.

[117] van Praag H., Fleshner M., Schwartz M. W., Mattson M. P. (2014). Exercise, energy intake, glucose homeostasis, and the brain. *Journal of Neuroscience*.

[118] Zhao W.-Q., Chen H., Quon M. J., Alkon D. L. (2004). Insulin and the insulin receptor in experimental models of learning and memory. *European Journal of Pharmacology*.

[119] Irving A. J., Harvey J. (2014). Leptin regulation of hippocampal synaptic function in health and disease. *Philosophical Transactions of the Royal Society B: Biological Sciences*.

[120] Mainardi M., Pizzorusso T., Maffei M. (2013). Environment, leptin sensitivity, and hypothalamic plasticity. *Neural Plasticity*.

